# Regulatory Mechanisms of Leaf Senescence in Herbaceous and Woody Perennials: A Comparative Review

**DOI:** 10.3390/plants15081248

**Published:** 2026-04-18

**Authors:** Wenliang Li, Juan Qi

**Affiliations:** Key Laboratory of Grassland Ecosystem of Ministry of Education, Pratacultural College, Gansu Agricultural University, Lanzhou 730070, China; liwenliang0928@163.com

**Keywords:** perennial plant, leaf senescence, regulatory mechanism, transcription, epigenetic, telomere systems

## Abstract

Leaf senescence in perennial species constitutes a highly orchestrated developmental phase that differs fundamentally from the obligate monocarpic senescence of annual plants. While individual organs undergo programmed senescence, prerennial organisms maintain longevity across multiple growing seasons through a sophisticated interplay between endogenous programs and exogenous cues. This review provides a systematic synthesis of the regulatory mechanisms governing leaf senescence in herbaceous perennials (*Lolium perenne* and *Festuca arundinacea*) and woody perennials (*Populus*, *Pinus*, and *Agave*). We highlight a multi-layered regulatory landscape, encompassing divergent and conserved pathways in transcriptional orchestration, hormonal crosstalk, metabolic reprogramming, and telomere maintenance. Specific emphasis is placed on how these mechanisms allow for tissue-specific and seasonal adaptation, such as the integration of dormancy signals in woody taxa versus stress-plasticity in perennial grasses. By elucidating these complex frameworks, this review not only advances our fundamental understanding of plant life-span regulation but also provides a theoretical foundation for the molecular breeding of delayed senescence germplasm, offering transformative potential for enhancing agricultural productivity and ecological resilience.

## 1. Introduction

In annual plants, leaf senescence is a strictly orchestrated and rapid developmental phase that concludes the life cycle [1]. Over recent decades, extensive research has elucidated the sophisticated, multi-layered regulatory networks governing senescence in model annuals such as *Arabidopsis thaliana* and *Oryza sativa* [1,2]. These findings have established a robust theoretical framework for genetic improvements in major food and oil crops [3,4,5]. However, the regulatory mechanisms in perennial species, which must balance annual organ senescence with systemic longevity across multiple years, remain comparatively unexplored. Elucidating these perennial-specific mechanisms is of profound scientific and practical implications, as they represent unique evolutionary adaptations to long-term environmental fluctuations. Senescence in perennial species is a highly orchestrated biological phase governed by hierarchical biomolecular networks [6]. As sessile organisms, perennials have evolved intricate developmental programs that coordinate endogenous signals, such as phytohormones and telomere dynamics, with exogenous environmental cues and epigenetic modifications [7,8,9]. A defining feature of perennials is the dynamic equilibrium maintained between programmed senescence, vegetative growth, and meristematic persistence, allowing for systemic longevity even as individual organs senesce [7]. By integrating genetic, metabolic, and environmental inputs, these plants optimally remobilize resources to sustain perennial vigor across successive growing seasons. This review specifically focuses on the contrasting and conserved mechanisms found in herbaceous perennials and woody perennials. While both groups utilize leaf senescence as a terminal stage of organ development and a critical form of programmed cell death (PCD), their strategies differ based on their ecological niches. In perennial woody models such as *Populus*, leaf senescence is fundamental to nutrient salvaging and storage for winter dormancy [10]. Conversely, in perennial grasses, senescence often exhibits higher plasticity, mediating rapid trade-offs between biomass accumulation and stress adaptation [11]. Consequently, the strategic manipulation and delay of senescence have emerged as key objectives for enhancing the productivity and quality of perennial crops, forage, and bioenergy feedstocks [12]. Furthermore, the precise regulation of senescence confers an adaptive advantage by enhancing resilience to both abiotic and biotic stresses, which is foundational to agricultural productivity and ecosystem sustainability [13]. Elucidating the multi-layered regulatory networks of perennial senescence is therefore essential for advancing our fundamental understanding of plant longevity and optimizing resource use efficiency.

## 2. Intrinsic Developmental Regulation of Leaf Senescence in Perennials

Senescence in perennial plants, while genetically programmed, is a highly plastic process. Its onset and progression are shaped by continuous interactions between endogenous developmental cues and fluctuating environmental conditions. Distinct from annuals, perennials must balance organ-level programmed cell death with the long-term maintenance of meristematic vitality to support polycarpic growth. At the core of this intrinsic regulation is an age-dependent genetic clock that initiates senescence once a leaf reaches a specific physiological threshold. In woody perennials such as Populus, primary research has identified the PtRD26-mediated transcriptional module as a key internal regulator. Transcriptomic profiling reveals that PtRD26 and its alternative splicing variants fine-tune the timing of autumn senescence by orchestrating a cascade of NAC transcription factors, ensuring that nutrient salvaging is completed before the onset of winter dormancy [14]. Similarly, in the perennial legume Medicago sativa, the stay-green trait is linked to the activity of specific transcription factors like SGR-associated regulators that overcome a major barrier in the utilization of genome editing for alfalfa improvement [15]. The intrinsic program also involves a dynamic shift in primary and secondary metabolism as a function of leaf age. In *Elymus sibiricus*, age-dependent leaf senescence is characterized by a significant decline in zeatin (ZT) levels and a concomitant increase in abscisic acid (ABA) concentrations, even under optimal growing conditions. These hormonal shifts represent an internal metabolic “count-down” that precedes any visible signs of chlorophyll degradation [16]. Furthermore, in certain perennial species like *Agave tequilana*, telomere-associated genes and telomerase activity provide an additional layer of intrinsic regulation, linking cellular aging and differentiation to the plant’s overall developmental clock.

This orderly, genetically programmed recycling is a vital strategy for nutrient remobilization, where macromolecules such as proteins, nucleic acids, and lipids are degraded, and the resulting nutrients are recycled to perennial storage tissues in the stems or roots to provide the essential material basis for growth in the following season. By synthesizing these primary research findings from diverse perennial models, it is clear that the internal developmental program is a robust, multi-dimensional system that defines the perennial life-history strategy.

## 3. Environmental Factors Regulate Leaf Senescence in Perennial Plants

Exogenous environmental factors modulate plant senescence through various physiological and molecular pathways. While properly timed senescence is essential for optimizing offspring survival and seasonal adaptation, premature senescence induced by environmental stress can severely compromise crop productivity and the quality of agricultural products [17]. In perennials, these environmental drivers often engage in complex synergistic or antagonistic crosstalk [18]. These include a range of biotic stresses, such as pathogen infection, insect herbivory, and animal trampling and abiotic stresses, including low or extremely high light intensity and water deficit or waterlogging [19]. This regulatory cascade initiates distinct physiological and molecular changes. A hallmark of leaf senescence is chloroplast degradation, which unmasks pre-existing carotenoids and is often accompanied by the independent accumulation of anthocyanins, leading to the characteristic shift from green to yellow or red hues [20,21]. This degenerative phase is characterized by organelle disintegration and substantial shifts in the metabolite profiles and gene expression [22,23]. Ultimately, these senescence-associated processes compromise photosynthetic capacity and biomass accumulation [24], thereby undermining both agricultural productivity and ecological fitness.

The sensing of environmental cues is the primary trigger for the initiation of leaf senescence in perennials [25]. To bridge the gap between ecosystem-level observations and cellular mechanisms, remote sensing indices such as the Plant Senescence Reflectance Index (PSRI), calculated as (R_680_ − R_500_)/R_750_, serve as vital nondestructive markers. By quantifying the carotenoid-to-chlorophyll ratio, PSRI effectively captures the early stages of pigment shift and chlorophyll degradation, providing a macro-phenotypic proxy for the onset of the senescence program under fluctuating light intensities or climatic shifts [26]. In perennial ecosystems, these phenological transitions are frequently driven by interannual variations in precipitation and temperature, which act as primary upstream triggers for molecular reprogramming. For example, heat stress significantly accelerates the senescence trajectory, a process that can be mitigated by modulating central metabolism. In perennial ryegrass, exogenous application of alpha-ketoglutarate (AKG) has been shown to significantly delay heat-induced leaf senescence by enhancing antioxidant scavenging, modulating amino acid and respiratory metabolism, and maintaining membrane stability [27]. *Prolonged* exposure to high temperatures substantially accelerates leaf senescence and negatively impacts plant growth and development [28,29]. Complementing these metabolic adjustments, the interplay between plant carbon source and sink dynamics, so that senescence occurs later upon low carbon inputs and earlier upon low carbon demand [30]. By enhancing nutrient availability and alleviating drought-induced oxidative stress, biochar sustains biomass accumulation in species like ryegrass [31]. Under drought conditions, biochar application significantly bolsters photosynthetic parameters and nutrient levels in perennial ryegrass leaves, leading to substantial biomass accumulation [32]. Similarly, micronutrient signaling, such as iron supplementation, reinforces antioxidant defenses in both salt-tolerant (*Agrostis scabra*) and salt-sensitive (*A. stolonifera*) grasses, primarily through the upregulation of protective isozymes and gene expression [33]. Ultimately, because the accumulation of oxidative damage is a central driver of aging, the superior longevity of perennial species is often attributed to their robust mechanisms for attenuating ROS. By maintaining cellular integrity through enhanced H_2_O_2_ scavenging and multi-dimensional signaling integration, perennials successfully navigate the trade-offs between seasonal stress adaptation and systemic survival across multiple growing seasons [34,35].

Leaf senescence in perennials is finely tuned by light and temperature cues, which signal seasonal transitions [36]. Beyond standard photoperiods, stress-level light conditions, including both ultraviolet (UV) radiation and low-light intensity, act as critical triggers for premature senescence [37]. For instance, UV-induced damage is mediated by specific microRNAs like miR164 in perennial ryegrass (*Lolium perenne*), which modulates electrolyte leakage and water content [38]. Similarly, prolonged low-light conditions accelerate leaf aging in Bermudagrass, thereby compromising turfgrass aesthetics and quality [39]. Conversely, atmospheric factors such as elevated CO_2_ levels can provide a mitigating effect by enhancing photosynthetic efficiency and total biomass [40]. However, under combined high-temperature and water-deficit stress, *Phalaris arundinacea* undergoes premature senescence and a reduction in aboveground biomass. Additionally, leaf spot fungus infection in alfalfa stimulates glycolysis and β-oxidation within the tricarboxylic acid cycle, accelerating senescence and ultimately leading to plant death [41]. In switchgrass, Panicle removal significantly increases chlorophyll content, net photosynthetic rate, and photochemical quantum efficiency in flag leaves; this is accompanied by a significant decrease in abscisic acid (ABA) levels in flag leaves and stems but increases in rhizomes, effectively delaying whole-plant senescence [42]. Amid ongoing industrial development and rising carbon emissions, atmospheric CO_2_ accumulation continues to influence perennial productivity [43,44]. Global warming and rising CO_2_ concentrations also promote canopy development in North American grasslands during spring, delay autumn senescence, and moderately enhance overall plant production [45]. In heavy metal-contaminated experimental fields within degraded ecosystems, Miscanthus leaves accumulate elevated concentrations of lead Pb and Zn. These accumulations suppress photosynthetic rates and antioxidant activity, leading to premature leaf senescence [46]. Similarly, under severe stress, lemongrass (*Cymbopogon* spp.) exhibits early senescence and chlorosis, posing a significant challenge to sustainable agricultural practices in contaminated regions [47]. Unlike annuals, perennials must balance these environmental responses with long-term survival and nutrient storage for the next growing season.

## 4. ROS and Enhanced Protease and Nuclease Activities Drive Leaf Senescence in Perennials

**Figure 1:**
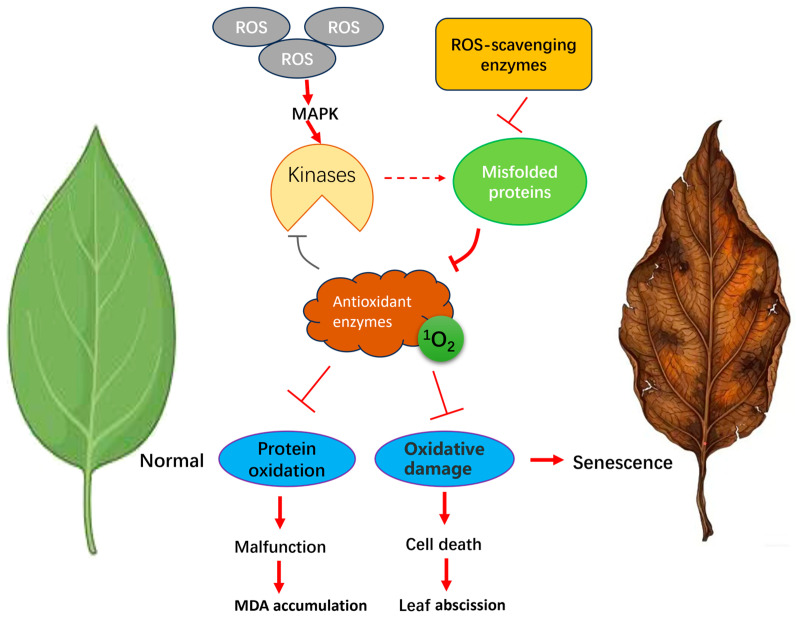
Regulatory network of reactive oxygen species (ROS) and antioxidant enzymes during leaf senescence in perennial plants. This schematic illustrates the dynamic interplay between ROS accumulation and antioxidant defense mechanisms that govern cellular aging. Solid arrows (→) denote direct activation pathways, whereas dashed arrows (⇢) represent indirect activation, potentially involving intermediate signaling molecules. T-bars (⊣) indicate inhibitory effects, highlighting negative feedback loops or the suppression of target pathways. The thickness of the lines corresponds to the magnitude or intensity of the regulatory flow, reflecting how signaling strength shifts across different physiological states or developmental stages of senescence. The figure was created using Bioicons (https://bioicons.com).

The interplay between reactive oxygen species (ROS) and antioxidant enzymes within the senescence network of perennial plants is depicted in Figure 1. ROS are oxygen-containing free radicals and peroxides derived from metabolic processes, and they play a central role in mediating perennial plant responses to biotic and abiotic stresses [48]. All plants possess ROS-generating systems, and strong correlations have been observed among plant age, ROS production levels, and oxidative tissue damage [29,49]. At homeostatic levels, ROS regulate key developmental processes such as cell proliferation, differentiation, programmed cell death, and organ senescence [50]. These biochemical changes, including ROS accumulation and enzymatic activities, are hallmark features of leaf senescence, facilitating nutrient remobilization in perennial species. However, excessive ROS accumulation is a major driver of cellular aging. This oxidative burden promotes fatty lipid and protein oxidation, induces DNA damage, and ultimately triggers cellular senescence, functional decline, and physiological dysfunction [51]. Chloroplasts are recognized as primary sites of ROS generation, where photosystems in thylakoid membranes predominantly produce singlet oxygen (^1^O_2_) and superoxide anions (O_2_^−^) [52]. While excessive ROS accumulation is detrimental to cells, a dynamic equilibrium between ROS production and scavenging critically regulates physiological and biochemical processes during plant senescence [48,53]. Recent studies indicate that maintaining ROS homeostasis through modulation of the TOR/SnRK1 signaling pathway effectively delays petal senescence in roses [54]. Additionally, ROS function as signaling molecules that activate senescence-associated transduction pathways and induce the expression of antioxidant enzyme genes [55,56]. Overexpression of ROS-scavenging enzymes significantly reduces oxidative stress, enhances plant tolerance, and delays senescence [57]. In *Zoysia japonica*, exogenous application of spermidine and spermine enhances leaf antioxidant activity, thereby alleviating cold-induced leaf senescence [58]. Similarly, in lemongrass under arsenic stress, enhanced ROS scavenging mitigates oxidative damage, thereby maintaining redox homeostasis and delaying the onset of senescence [47]. The efficient scavenging of ROS is essential for attenuating oxidative stress and preserving the functional integrity of organelles during senescence. The subsequent upregulation of proteases and nucleases facilitates the remobilization of nutrients from senescing leaves to perennial storage organs, supporting overwintering and regrowth in the following season. This strategic nutrient recycling is a defining feature that distinguishes leaf senescence in perennials from the terminal senescence observed in annual plants [34].

## 5. Hormonal Imbalance as a Key Driver of Perennial Plant Senescence

**Figure 2:**
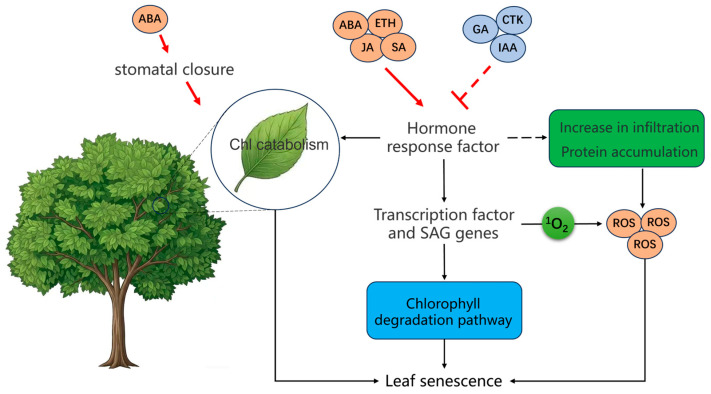
Regulatory networks of plant hormones and transcription factors during leaf senescence. This schematic illustrates the complex signaling cascades and transcriptional reprogramming that govern the progression of leaf senescence. It highlights the dynamic interplay, both synergistic and antagonistic, between senescence-promoting phytohormones and senescence-delaying hormones. Furthermore, it depicts how these hormonal signals are transduced to activate or repress specific families of transcription factors and regulators orchestrating the expression of downstream senescence-associated genes (SAGs). The figure was created using Bioicons (https://bioicons.com). Solid arrows indicate activation, dashed arrows indicate indirect effects, and T-bars represent inhibition.

Phytohormones and growth regulators are fundamental to orchestrating leaf senescence [59,60]. Environmental stress signals trigger dynamic changes in endogenous hormone levels, which are integrated into developmental programs through intricate regulatory networks [5]. As illustrated in Figure 2, these signaling molecules play critical roles throughout the progression of senescence. Based on current understanding, plant hormones can be categorized as either senescence-promoting or senescence-inhibiting (Table 1). Specifically, Ethylene, jasmonic acid (JA), salicylic acid (SA), abscisic acid (ABA), brassinosteroids (BRs), and strigolactones (SLs) act as senescence promoters, whereas auxins (IAA), cytokinins (CTKs), and gibberellins (GAs) function as senescence inhibitors [61,62]. During vegetative growth, hormones synthesized in both aerial and subterranean tissues are transported and engage in feedback regulation, thereby coordinating their biosynthesis to sustain normal growth and metabolism [63,64].

In *Elymus sibiricus*, leaf senescence is closely linked to oxidative stress and is characterized by reduced zeatin (ZT) levels coupled with elevated ABA concentrations [16]. Exogenous application studies in tall fescue (*Festuca arundinacea*) demonstrate that both ABA and SA induce characteristic senescence symptoms, such as leaf chlorosis, reduced biomass, increased proline accumulation, and enhanced electrolyte leakage [64]. In poplar, SA exerts a complex regulatory role in senescence and programmed cell death (PCD). The SA signaling pathway exhibits intricate crosstalk with ROS metabolism, glycosylation, and the unfolded protein response (UPR), which can either alleviate endoplasmic reticulum (ER) stress or promote cell death depending on the duration of activation. Consequently, SA can either suppress ROS production or potentiate ROS signaling based on the physiological context [65]. In *Zoysia japonica*, leaves initiate senescence after approximately four months of growth, coinciding with a 41% decrease in IAA content. This decline reflects the inhibition of IAA biosynthesis alongside the activation of IAA signaling pathways. In contrast, ABA levels increase by 190%, indicating that ABA signaling is strongly activated during leaf senescence in *Z. japonica* [66]. Similar hormonal dynamics occur in *Borderea pyrenaica*, where leaf senescence is associated with a pronounced reduction in IAA and an increase in ABA [67]. Beyond its role in senescence, ABA is central to stomatal regulation; under severe environmental stress, it limits transpirational water loss and reprograms metabolism to favor the accumulation of compatible solutes, ultimately accelerating leaf senescence as an adaptive survival strategy [68].

## 6. Transcriptomic Networks and Key Genes Regulating Leaf Senescence in Perennial Plants

A growing body of evidence indicates that leaf senescence-associated transcription factors (TFs) serve as pivotal regulators in the initiation and progression of the senescence program [69], which is governed by a complex regulatory network (Table 2). Recent advances in omics technologies, particularly transcriptomics, have significantly enhanced our understanding of these regulatory mechanisms and provided critical insights into the transcriptional orchestration of orderly leaf senescence [70,71]. Transcriptional regulation is a central component of this network, in which TFs precisely control the spatiotemporal expression of senescence-associated genes (SAGs) to trigger and drive leaf senescence [18]. Acting upstream of the leaf senescence regulatory pathway, TFs activate various senescence-related genes (Figure 2). Among these, members of the NAC and WRKY families play particularly vital roles. In European aspen (*Populus tremula*), the core transcriptional responses during autumn leaf senescence involve two tightly coordinated processes: the repression of chloroplast functions and induction of stress-defense responses. These processes are largely mediated by NAC and WRKY TF families, which are widely implicated in leaf senescence regulation across plant species. Notably, NAC100 and WRKY75 have been identified as key regulatory TFs that are consistently upregulated during leaf senescence in diverse deciduous tree species [65]. NAC transcription factors are central regulators of leaf senescence, with key members ORE1/ANAC092 negatively regulated by miR164 to fine-tune senescence progression [72,73]. This mechanism has been documented in Arabidopsis and shows promising associations in sunflower [74,75,76]. In lychee, thirteen senescence-associated *LcNAC* genes are differentially expressed during both natural and low-temperature-delayed senescence. Moreover, LcMYC2 directly binds to the LcNAC1 promoter, positively regulating its expression and contributing to ABA-mediated fruit senescence [77]. In perennial ryegrass, analysis of a truncated promoter linked to the stay-green trait (LpSGR) identifies the NAC family TF LpNAL as a central regulator of chlorophyll catabolism. Overexpression of LpNAL results in delayed leaf senescence or stay-green phenotypes, whereas RNA interference (RNAi)-mediated knockdown accelerates leaf senescence [78]. A significant overlap exists between the transcriptomic profiles of long-term heat stress and senescence, including the upregulation of chlorophyll catabolism genes, hormonal metabolic genes, and NAC TFs. In addition, extensive alternative splicing (AS) has been documented in genes responsive to both heat stress and senescence. Emerging evidence suggests that AS serves as a critical post-transcriptional regulatory layer in the regulation of leaf senescence. For instance, the differential splicing of key transcription factors, such as members of the NAC families, has been shown to modulate the onset and progression of the senescence program. This mechanism allows perennial plants to rapidly adjust their physiological responses to fluctuating environmental signals [18,79]. While short-term heat stress can activate protective gene expression, prolonged heat stress functions as a damaging factor that accelerates leaf senescence [28]. In tall fescue, FaNAC047 accelerates heat-induced leaf senescence by exacerbating photosystem damage and promoting ROS accumulation, while its knockout delays the process [29]. WRKY proteins regulate SAGs either directly, by binding to W-box elements in promoter regions, or indirectly, by regulating hormone-related genes [80]. In perennial ryegrass, LpWRKY69 and LpWRKY70 are transcriptionally activated during heat-induced leaf senescence and are further modulated by hormonal signals [81].

## 7. Protein Turnover and Metabolic Regulation During Leaf Senescence in Perennial Plants

Programmed cell death (PCD) during plant leaf senescence is fundamentally linked to coordinated protein hydrolysis, a process that is particularly critical in perennials for seasonal nutrient salvaging [82]. Unlike annuals, perennials must execute high-efficiency protein disassembly to relocate nitrogen and minerals from senescing leaves to long-term storage organs (woody stems or rhizomes) to support future growth cycles [83]. The accumulation of oxidative damage, coupled with impaired protein biosynthesis, including increased protein misfolding and reduced translational elongation efficiency, triggers the degradation of protein aggregates, which acts as a key molecular driver of accelerated plant senescence [35].

**Figure 3:**
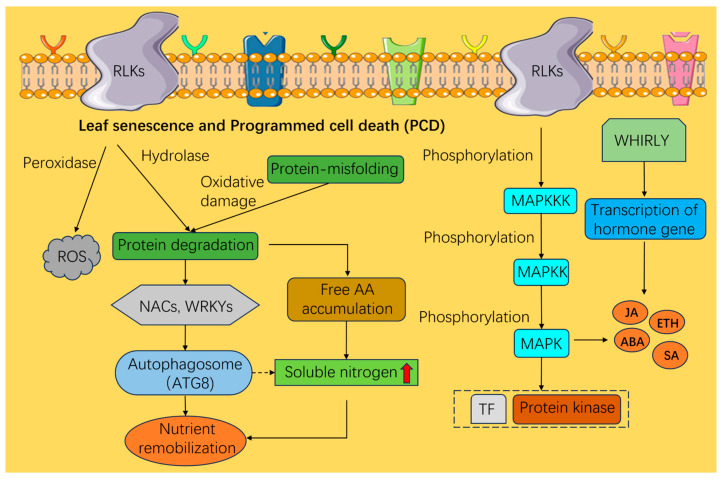
Protein regulatory network governing the leaf senescence process in perennial plants. It highlights post-translational modifications and protein–protein interactions that orchestrate the stabilization of specific senescence-associated proteins. Arrows delineate physical interactions, enzymatic modifications, or degradation signaling pathways. The figure was created using Bioicons (https://bioicons.com). Solid arrows indicate activation, dashed arrows indicate indirect effects.

Recent advances in mass spectrometry and computational biology have significantly improved proteome-wide mapping of protein–protein interactions, thereby accelerating progress in the field of plant biological research [84]. Reversible protein phosphorylation, mediated by protein kinases and phosphatases, plays a central role in regulating senescence in perennial plants [85]. In perennial models such as Populus, recent proteomic analyses have identified specific senescence-associated proteases (SAPs) that are transcriptionally upregulated during autumn [86]. Receptor-like kinases (RLKs) and MAPK cascades, while conserved, exhibit perennial-specific regulatory roles in sensing seasonal cues. In woody perennials, specific Leucine-Rich Repeat RLKs (LRR-RLKs) have been implicated in perceiving the transition from active growth to senescence [87]. While much of our understanding of RLK-mediated signaling is extrapolated from Arabidopsis, studies in *Populus tremula* suggest that certain RLKs coordinate with cell-to-cell communications to initiate the signals during plant secondary growth and wood formation [88]. This phosphorylation-dependent signaling ensures that protein degradation occurs in an orderly fashion, preventing premature cell death before nutrient recycling is complete [89,90]. In addition to the signaling pathways mediated by membrane-bound RLKs, which perceive extracellular cues, senescence is also regulated by distinct families of proteins such as WHIRLY proteins. Unlike the kinase-based signaling of RLKs, WHIRLY proteins act as dual-localized DNA-binding proteins in plastids and the nucleus, where they modulate the expression of senescence-associated genes through retrograde signaling. Furthermore, WHIRLY proteins act as multifunctional regulators that modulate both developmental and stress-induced senescence by interacting with DNA/RNA and proteins within organelle nucleoids and the nucleus (Figure 3). In Agave and other long-lived species, WHIRLY1 facilitates retrograde signaling between plastids and the nucleus, modulating the expression of SAGs during the extended leaf lifespans characteristic of these taxa [5]. Furthermore, the mitogen-activated protein kinase (MAPK) cascade provides a hierarchical signaling scaffold. In perennial ryegrass (*Lolium perenne*), the activation of specific MAPK modules has been linked to the phosphorylation of senescence-associated transcription factors. LpMPK6 suppresses LpMYBR1-mediated transcriptional activation of LpNRT1.5 via phosphorylation, thereby regulating NO_3_^−^ transport and leaf coloration in perennial ryegrass [91]. This link between kinase signaling and autophagy is a crucial mechanism that allows perennials to maintain protein homeostasis under fluctuating environmental stresses while preparing for dormancy [92,93].

## 8. Epigenetic Reprogramming During Senescence in Perennial Plants

Perennial plant senescence involves extensive reprogramming of gene expression across multiple regulatory layers, where epigenetic mechanisms provide a “molecular memory” to balance annual organ turnover with systemic longevity [94]. Increasing evidence indicates that epigenetic reprogramming is a major coordinator of cellular aging in perennial plants [95,96]. This process is characterized by heritable and dynamic changes in the chromatin state, including DNA methylation, histone modifications, chromatin remodeling, non-coding RNAs, and RNA methylation [97].

**Figure 4:**
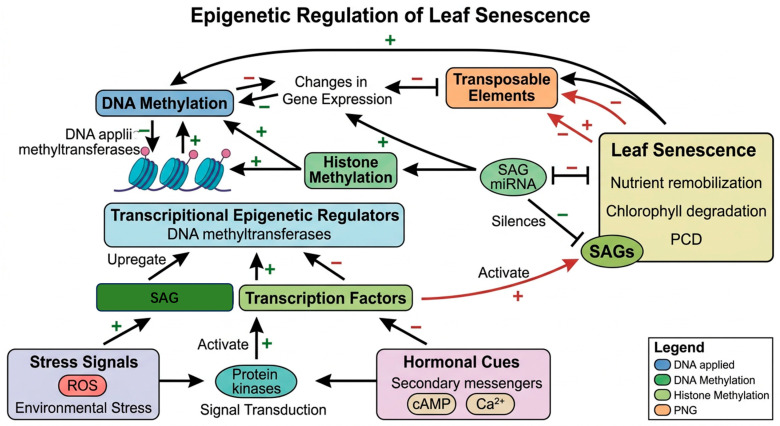
Multi-layered epigenetic regulation mechanism of leaf senescence in perennial plants. The integration of these epigenetic layers ensures the precise temporal and spatial execution of the leaf senescence program. The figure was created using Bioicons (https://bioicons.com). Solid arrows indicate activation, the blue circles with a purple dot represent transferases.

Unlike annuals, perennials utilize specific histone marks to gate the seasonal expression of senescence-associated genes (SAGs). In Populus species, the onset of autumn senescence is tightly regulated by the repressive histone mark H3K27me3 [98]. The synchronized removal of these marks in response to shortening photoperiods allows for a rapid and coherent induction of the senescence program across the entire canopy, a mechanism that ensures nutrient recovery before winter dormancy [99]. In addition to histone modifications, DNA serves as a conserved epigenetic rheostat in perennial [100]. For instance, arsenic accumulation reduces DNA methylation levels in the fronds of *Pteris ferns* and decreases chlorophyll content, indicating that DNA methylation modulates photosynthesis and pigment accumulation under arsenic-induced stress [101]. Significant differences in DNA methylation are also observed in the meristematic tissues of juvenile and mature *Pinus radiata* at different developmental stages, whereas these differences are less pronounced in differentiated tissues. Notably, as the degree of rejuvenation increases, global genomic DNA methylation in meristematic regions gradually declines [102]. Specifically, the hypermethylation of specific promoter regions in mature Pinus tissues prevents the premature activation of senescence pathways, a stark contrast to the rapid epigenetic decline seen in annual models. WHIRLY proteins, as single-stranded DNA- and RNA-binding proteins with dual localization in the nucleus and plastids, function as key transcriptional and epigenetic regulators during leaf senescence (Figure 4). Emerging evidence suggests a dynamic regulatory feedback loop between oxidative stress and genome stability. The accumulation of ROS acts as a critical signal that modulates the activity of Transposable Elements (TEs). Under normal physiological conditions, TEs are maintained in a transcriptionally silent state via epigenetic mechanisms, primarily DNA methylation and histone modification [103,104]. However, senescence and environmental stress lead to excessive ROS accumulation, which can impair the maintenance of these repressive epigenetic marks. High levels of ROS have been shown to induce global hypomethylation and chromatin decondensation, thereby “releasing” TEs from their silenced state. This reactivation allows for TE mobilization and transcriptional upregulation [95,98]. In the context of senescence, this relationship forms a self-amplifying cycle. The initial ROS burst triggers TE activation: subsequent retrotransposition events and the resulting genomic instability can trigger a DNA damage response (DDR), which further stimulates ROS production. This ROS-TE-DDR axis accelerates the PCD characteristic of leaf senescence [34]. In perennial species, WHIRLY proteins play a dual role here: they act as single-stranded DNA-binding proteins that stabilize the genome in both the nucleus and plastids, while also modulating SAG expression through retrograde signaling [5]. These complex epigenetic regulatory networks allow perennial plants to finely tune gene expression throughout their decade or century-long life cycles, responding to environmental cues while orchestrating transitions between physiological states. Future research utilizing ATAC-seq and single-cell epigenomics in perennial models will be essential to further elucidate how these distinct modifications are coordinated during the senescence transition [18].

## 9. Chromosomal Telomeres Are Strongly Linked to Plant Senescence

**Figure 5:**
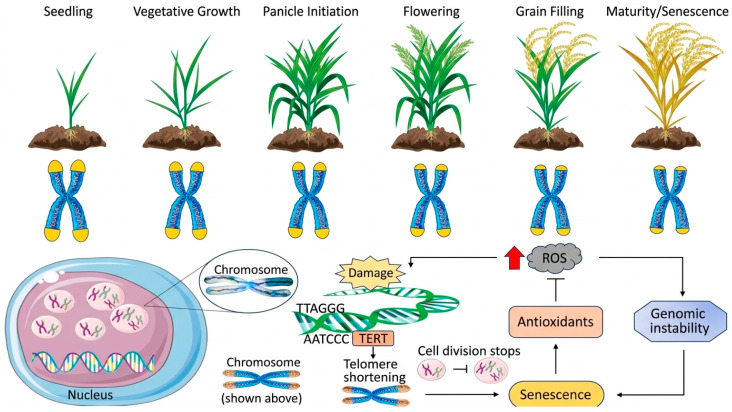
Molecular regulation mechanisms of chromosome telomeres during leaf senescence in perennial plants. Highlights the balance between telomere shortening driven by the end replication problem and environmental stressors such as reactive oxygen species and telomere elongation mediated by the telomerase ribonucleoprotein complex, including TERT and TR components. Demonstrates how critical telomere shortening triggers a DNA Damage Response, leading to cell cycle arrest and the subsequent induction of senescence-associated pathways. The figure was created using Bioicons (https://bioicons.com). *‘Solid arrows indicate promote, red arrows indicate rise, T-bars (⊣) represent inhibition’*.

Among the various theories of plant aging, the telomere theory is widely accepted due to its significant explanatory power. Telomere dysfunction is a primary driver of age-related decline, largely because DNA polymerases are unable to completely replicate the termini of linear chromosomes [105,106]. With each cell division, terminal telomeric repeats are progressively lost with each cell division, compromising telomere length and sequence integrity, which in turn accelerates the aging process [107]. Telomere attrition underscores the inherent limitation of DNA polymerases in fully replicating eukaryotic chromosome ends [108,109]. After multiple rounds of cell division, critically shortened telomeres trigger genomic instability, ultimately resulting in apoptosis or cellular senescence. Notably, phytochemicals can function as telomerase activators, directly limiting telomere shortening through antioxidant activity, and indirectly attenuating telomere attrition (Figure 5).

Research on the regulation of telomere length and telomerase activity in plant leaf senescence remains limited, with studies primarily focused on model species and major crops such as *A. thaliana* [110]. Telomerase is an active ribonucleoprotein complex that extends telomeres to maintain their length and mitigate the deleterious effects of telomere attrition. The presence and activity of telomerase establish a dynamic equilibrium, allowing fluctuations in telomere length within a defined range [111]. In *E. sibiricus*, telomerase activity is primarily determined by plant age and developmental stage. This activity declines across various tissues and organs as the plant ages, though it remains relatively high during the jointing stage [112]. Consistently, telomere length decreases continuously during the milk maturity period as the age of the plants increases. Moreover, it was observed that telomerase activity gradually declined with the increasing age of the plants [113]. Unlike annual plants, which undergo obligate monocarpic senescence (a programmed death following a single reproductive cycle), perennial plants exhibit a highly coordinated senescence process that occurs at both the organ and organismal levels (Table 3). This allows perennials to maintain longevity through multiple growing seasons. This tissue-specific maintenance is a key feature that supports the long life-span of perennials. In *Pinus longaeva*, measurements of telomerase activity and telomere length in leaves from trees of different ages revealed a close relationship between tree age, telomere dynamics, and telomerase activity [114]. Studies in the perennial *Agave americana* further indicate that telomerase is associated with cellular elongation, division, and differentiation [115]. Telomere length is closely linked to cellular senescence and metabolic status, and exhibits marked interspecific and intraspecific variation in plants [116,117]. However, some studies suggest that the expression of telomerase alone does not ensure telomere extension. This is because telomeric TTAGGG repeats can form stable four-stranded G-quadruplex structures that act as physical barriers, impeding telomerase access to telomeric DNA [118]. Variation in telomerase activity has been systematically characterized across different tree ages, growing seasons, tissue types, and sexes. Telomerase activity peaks during periods of vigorous growth and at elevated temperatures, whereas it declines during dormancy; notably, activity remains highest in meristematic tissues [119,120]. Despite these findings, investigations into the correlation between telomere length and telomerase activity have yielded inconsistent results, and a unified model has yet to emerge. Future research aimed at modulating the longevity of perennial plants should prioritize the study of telomere-associated genes and their regulatory mechanisms. Drawing parallels from animal systems, it is essential to determine—potentially through genome-wide association studies (GWAS)—whether specific single-nucleotide polymorphisms underlie inter-individual variation in telomere length.

## 10. Modern Methodological Approaches in Perennial Leaf Senescence Research

The study of leaf senescence in perennials has evolved from descriptive physiological observations to systemic molecular dissections, driven by the integration of omics and genome editing technologies. Metabolomics has become indispensable for tracing the massive nutrient remobilization that characterizes perennial senescence [121]. Unlike annuals, perennials must strategically manage carbon and nitrogen flux to support overwintering and subsequent spring budburst [122]. Recent metabolomic profiling has revealed that specific shifts in primary metabolites, such as *LpNOL*, could regulate heat-induced leaf senescence in perennial ryegrass through metabolic reprogramming in the pathways of respiration, secondary metabolism, antioxidant metabolism, and protein synthesis [123]. Complementing these metabolic insights, proteomics addresses the functional execution of senescence programs that transcriptomic data alone may not fully capture (Table 4). In perennials, protease inhibitors regulate various proteins that may be involved in reducing heat-induced leaf senescence and improve the heat resistance of *Creeping bentgrass* by reducing heat-induced proteolysis and maintaining the integrity of protein [124].

The application of CRISPR/Cas9-mediated genome editing has revolutionized the study of leaf senescence by transitioning the field from correlative transcriptomic observations to definitive causal validation [125]. In perennial species, which often face challenges such as long juvenile phases and complex polyploidy, CRISPR/Cas9 provides a powerful tool to bypass the limitations of traditional breeding and chemical mutagenesis. Specifically, this technology has been extensively utilized to knock out core Senescence-Associated Genes (SAGs), particularly those encoding NAC and WRKY transcription factors that act as master regulators of the senescence onset [126]. For instance, targeted mutagenesis of the NAC gene family in various perennial models has demonstrated a significant “stay-green” phenotype, effectively decoupling the chronological age of the leaf from the initiation of chlorophyll degradation [127]. Beyond simple gene knockouts, recent advancements such as CRISPR activation (CRISPRa) and base editing allow for the fine-tuning of senescence-related signaling pathways, such as the ethylene response networks [128]. By precisely modulating the promoter regions of genes involved in nutrient remobilization, researchers can now engineer perennial plants that maintain high photosynthetic efficiency for longer durations while still ensuring efficient nitrogen salvage before winter dormancy. This dual capability functional gene discovery and the creation of elite germplasm with enhanced biomass positions CRISPR/Cas9 as a cornerstone of modern perennial plant research [129].

## 11. Conclusions and Future Work

Perennial plant leaf senescence is an intricately orchestrated developmental phase, governed by the systemic integration of multi-layered regulatory networks that distinguish it from the obligate, terminal senescence of annual plants. While our understanding of these processes has historically been grounded in annual models like Arabidopsis, this review synthesizes recent evidence to propose a tri-tiered hierarchical model specifically tailored to the unique life-history strategies of herbaceous and woody perennials.

The Epigenetic and Genomic Stability Tier: At the foundational level, perennials utilize epigenetic memory to balance annual organ turnover with systemic longevity. Mechanisms such as PRC2-mediated H3K27me3 gating documented in woody models like Populus ensure that senescence-associated genes (SAGs) remain silent during the juvenile phase or active growing seasons. Furthermore, the ROS-TE-DDR axis serves as a critical checkpoint; maintaining transposable elements (TEs) in a silent state via DNA methylation is essential for preserving genomic integrity across decades or centuries of environmental exposure.

The Signaling and Proteolytic Tier: At the intermediate level, signal transduction components, including RLKs and MAPK cascades, are recalibrated for high-efficiency nutrient remobilization. Unlike the death-oriented proteolysis in annuals, perennials coordinate protein disassembly with seasonal dormancy cues, allowing essential resources to be salvaged and stored in perennial structures for subsequent growth cycles. WHIRLY proteins further integrate these signals by acting as dual-localized regulators between organelles and the nucleus.

The Metabolic and Phytohormonal Tier: At the executive level, perennials exhibit high physiological plasticity. The dynamic equilibrium between senescence-inhibiting and promoting hormones is fine-tuned by key metabolites such as α-ketoglutarate (AKG). This tier allows perennial grasses like *Lolium perenne* to rapidly adjust their senescence trajectory in response to recurring abiotic stresses while maintaining overall plant vigor. Despite these insights, the regulatory landscape of perennial leaf senescence remains fragmented. Future research must transcend the identification of isolated components and move toward dissecting the complex networks that integrate these layers.

Specifically, three pivotal challenges remain:

1. Deciphering Seasonal Integration: How do perennials integrate short-term stress signals with long-term seasonal clocks at the epigenetic level to prevent premature senescence?
2. Optimizing the Sink-Source Transition: Can molecular design and gene editing be used to extend the “green-window” of photosynthesis in bioenergy perennials without compromising the nitrogen salvaging required for next year’s regrowth?
3. Cross-Taxa Functional Validation: To what extent are the regulatory hubs identified in herbaceous perennials conserved in woody deciduous models, and how do these differences dictate their respective life-history strategies?

Elucidating this multi-layered framework not only advances our fundamental understanding of plant longevity but also provides a strategic roadmap for enhancing agricultural productivity and ecological resilience in a changing climate. Addressing these questions is essential for the transition toward sustainable perennial-based agriculture.

## Figures and Tables

**Table 1:** Phytohormones regulatory roles in leaf senescence of perennial species.

Hormone Type	Class	Species	Experimental Approach	Key Findings	Ref
ABA	Promoting	*Elymus sibiricus*	Endogenous level measurement	Increased ABA levels are closely linked to oxidative stress and senescence.	[16]
ABA	Promoting	*Festuca arundinacea*	Exogenous application	Induces leaf chlorosis, reduces biomass, and increases electrolyte leakage.	[64]
SA	Promoting	*Festuca arundinacea*	Exogenous application	Promotes characteristic senescence symptoms and proline accumulation.	[64]
SA	Dual Role	*Populus tremula*	Pathway analysis	Exhibits complex crosstalk with ROS and UPR; it can alleviate ER stress or promote PCD.	[65]
IAA	Inhibiting	*Zoysia japonica*	Endogenous level measurement	Senescence coincides with a 41% decrease in IAA due to biosynthetic inhibition.	[66]
IAA	Inhibiting	*Borderea pyrenaica*	Endogenous level measurement	Senescence is associated with a pronounced reduction in IAA levels.	[67]
CTKs (ZT)	Inhibiting	*Elymus sibiricus*	Endogenous level measurement	Reduced zeatin (ZT) levels are a hallmark of leaf senescence initiation.	[16]

**Table 2:** Key senescence-associated TFs and their regulatory functions in various species.

TF Family	Gene Name	Species	Function	Target Genes	Ref
NAC	*NAC100*	*Deciduous trees*	Activator	Stress-defense & senescence	[65]
NAC	*LcNAC1*	*Litchi chinensis*	Activator	ABA-mediated fruit senescence; Bound by LcMYC2	[77]
NAC	*LpNAL*	*Perennial ryegrass*	Repressor (Stay-green)	Regulates *LpSGR* (chlorophyll catabolism)	[78]
WRKY	*WRKY75*	*Deciduous trees*	Activator	Core transcriptional response in autumn	[65]
WRKY	*LpWRKY69/70*	*Perennial ryegrass*	Activator	Heat-induced senescence; hormonal signaling	[81]
MYC	*LcMYC2*	*Lychee*	Activator	Binds to LcNAC1 promoter	[77]

**Table 3:** Summary of telomere dynamics and telomerase activity in various plant species.

Species	Plant Type	Telomerase Length/Dynamics	Tissue/Stage	Key Findings	Ref
*Arabidopsis thaliana*	Annual	Progressive loss per division	Present in meristems	Basic model for telomere attrition and senescence	[110]
*Elymus sibiricus*	Perennial	Decreases during mature period	Various tissues; Jointing to maturity	Telomerase activity is primarily determined by plant age and stage	[112,113]
*Pinus balfouriana*	Perennial	Correlated with tree age	Leaves; Various tree ages	Close relationship between tree age, telomere dynamics, and telomerase activity	[114]
*Agave americana*	Perennial	Linked to metabolic status	Elongating/differentiating cells	Telomerase is crucial for cellular elongation and differentiation	[115]
*Ginkgo biloba*	Perennial	Interspecific variation	Meristematic tissues; Growing season	Activity remains highest in meristems; affected by temperature/dormancy	[119,120]

**Table 4:** Comparison of Modern Research Methods in Perennial Leaf Senescence.

Method	Target Level	Primary Contribution
Proteomics	Proteins	Mapping signaling pathways and functional execution
Metabolomics	Small Molecules	Reflecting real-time physiological and metabolic states
CRISPR Editing	DNA/Genome	Validating gene function and establishing causality

## Data Availability

No new data were generated for this work.
